# Success and complication rates of non-precious alloy telescopic crowns in a general dental practice

**DOI:** 10.1007/s00784-023-05350-2

**Published:** 2023-11-01

**Authors:** R. J. Wierichs, E. J. Kramer, H. Meyer-Lueckel, S. Abou-Ayash

**Affiliations:** 1 Department of Restorative, Preventive and Pediatric Dentistry, School of Dental Medicine, University of Bern, Freiburgstrasse 7, CH-3010 Bern, Switzerland; 2 Private practice, Norden, Germany; 3 Department of Reconstructive Dentistry and Gerodontology, School of Dental Medicine, University of Bern, Bern, Switzerland

**Keywords:** Longevity, Risk factors, Telescopic crown, Severely reduced dentition, Removable partial dentures, Clinical study, Retrospective study

## Abstract

**Objectives:**

This retrospective, single-center, practice-based cohort study aimed to analyze factors associated with the success of removable partial dentures retained by telescopic crowns (TRPD).

**Materials and methods:**

TRPD which were placed in a single practice of a practice-based research network were analyzed. Data from 139 patients (age (SD): 66 (11) years; 66 female) with 174 TRPD including 488 non-precious alloy telescopic crowns (TC) between 2004 and 2016 were included. TC without any technical complication were considered as successful, and as survived, if they were still in function at the last check-up. Multilevel Cox proportional hazard models were used to evaluate the association between clinical factors and time until failure.

**Results:**

Within a mean follow-up period (SD) of 4.2 (3.3) years (min–max: 1 day–12 years), 372 (76%) TC (AFR_5years,TC-level_: 5.0%) as well as 136 (87%) TRPD (AFR_5years,TRPD-level_: 5.1%) (“worst-case scenario”) and 150 (86%) TRPD (AFR_5years,TRPD-level_: 3.4%) (“best-case scenario”) were considered as successful. The main failure types were recementation (*n* = 39), endodontic treatment (*n* = 36), and extraction (*n* = 35). TC in male patients showed 1.6 times higher risk for failure than in female patients (95%CI: 1.1–2.4; *p* = 0.023). TC on premolars showed 2.2 times higher risk for failure than on incisors (95%CI: 1.1–5.0; *p* = 0.023) and TC in dentures with ≤ 3TC showed 2.1 times higher risk for failure than TC in dentures with > 3TC (1.3–3.4; *p* = 0.042). Furthermore, TC on the most distal tooth in an arch showed 2.4 times higher risk for failure than TC on a more mesial tooth (1.5–3.8; *p* < 0.001).

**Conclusion:**

For removable partial dentures retained by telescopic crowns, high success rates could be found after up to 12 years. Patient-level and tooth-level factors were significantly associated with failure.

**Clinical relevance:**

For removable partial dentures retained by telescopic crowns, high success rates could be found after up to 12 years. Patient-level and tooth-level factors were significantly associated with failure.

## Introduction

In an aging world population, dental care and the knowledge regarding prevention of caries are improving, resulting in more retained teeth than past generations had [1]. Furthermore, the rate of edentulism among the elderly is continually decreasing [2]. However, for partially dentate patients, the use of non-precious alloy double crowns has increased in recent years [3, 4]. Designing RPD is still often difficult for clinicians. Various factors should be considered for the prosthetic restoration of these patients, e.g., prosthetic restoration preferences, economic aspects, number and locations of residual teeth, extraction, strategic implant placement, or the type of opposing dentition [5]. Particularly with a limited residual dentition, positioning the clasps without esthetic impairment is hardly possible [6]. Due to the latter, for patient with few remaining teeth, RPD retained by telescopic crowns (TRPD) can be a successful treatment option to conventional RPD [7–9].

Telescopic crowns (TC) represent a specific double crown type with parallel or nearly parallel walled (0–2°) primary crowns. Primary or inner crowns are cemented on the abutment teeth while the secondary or outer crowns are incorporated within the TRPD. Due to the parallelism of the contact surfaces of the primary and secondary crowns, firstly, retention force is created to keep the TRPDs in position [10] and, secondly, TRPD are supposed to preserve the abutment teeth [9]. Furthermore, TRPD are supposed to show a high oral comfort, satisfactory esthetics, and oral hygiene is relatively easy to perform [3, 7–9]. However, the described advantages may be offset by the disadvantages of high treatment costs and treatment requirements within the denture’s function [11].

Depending on the different types of double crowns, cumulative survival rates differed between 79 and 95% for frictional telescopic systems after 5–7 years [3, 12, 13], 67–94% for telescopic systems with resilience after 5 years [14], and 53.3–97% for conus crowns after 3 years [15]. Furthermore, when differentiating between rigid and non-rigid telescopic double-crowns, cumulative survival rates differed between 69–95% and 34–94% for the rigid and the non-rigid types after 5 to 10 years, respectively [5]. However, these data are based on double crowns, made from precious-alloys, and may be different if the telescopic crowns made of non-precious alloys. Clinical data on TRPDs retained by non-precious alloy TC is limited. Furthermore, most of the studies on TC have been university-based studies. Although these studies may allow a more standardized data collection with regard to exposures, confounders, and endpoints, such data might not reflect the effectiveness of daily dental care in general practice [16, 17]. Contrastingly, in general practice, dentist-, patient-, tooth-, and material-level factors together influence the results. To resolve this, studies require a large number of restorations in one dataset [17]. Practice-based studies offer the opportunity to (routinely) collect large datasets [18]. Furthermore, practice-based studies allow to better understand the impact of daily treatment decisions on restoration success in general dental practice [18].

Thus, the aim of the present retrospective, single-center, practice-based cohort study was, firstly, to evaluate the longevity of different removable partial dentures retained by non-precious alloy telescopic crowns and, secondly, to analyze factors influencing the success of these dentures after up to 12 years.

## Materials and methods

### Study design

This study was a retrospective, non-interventional, practice-based, clinical study. Reporting follows STROBE guideline for cohort studies. The study has been registered in the German Clinical Trials Register (DRKS-ID: DRKS00026937). Assessment of the status of the restorations was done when patients attended for routine care or recall. According to guidelines for good clinical practice (Clinical trials – Directive 2001/20/EC) [19], the European guidelines for good clinical practice (CPMP/ICH/135/95) [20], and a previous study [18], this study was, therefore, not subject to Medical Ethical Committee approval.

### Restoration selection

Dentists participating in a German dental practice-based research network (Arbeitskreis Zahnärztliche Therapie) were asked to participate. Two dentists in one private practice agreed to search their anonymized electronic patients’ files for the presence of TC. Inclusion criteria were as follows:

- Patient receiving removable partial dentures (RPD) solely retained by non-precious alloy telescopic crowns with friction fit (TRPD)

Exclusion criteria were as follows:

- Patient receiving RPDs with additional retention elements other than TC
- Patient receiving implant-borne dentures retained by telescopic crowns (TRPD).

### Data extraction

The following data were collected anonymously (without reference to patient names) from the electronic patient files of one (dental) practice management program (DS-WIN, Dampsoft GmbH – Die Zahnarzt-Software, Damp, Germany) ([21]):

On practice-level; dentist;

on patient-level; age, sex, date of the first insertion of the TC, date of any (second) restorative (re-)intervention, date of the last visit, number of teeth/restorations (per patient/per the jaw in which the TRPD was worn) being included in the study, number of the regular check-ups and overall visits (including visits for check-ups, planned non-, micro-, or invasive treatments or emergency care), insurance status (private or statutory), DMFT.

on patient’s tooth-level; type of denture support (point-like, linear, triangle, rectangle), number of abutment teeth (≤ 3 vs. > 3), characteristics of the involved tooth (Fédération Dentaire Internationale [FDI] notation system), mode of failure, type of restoration.

### Success and failure of treatment

The observation period started with the TRPD being placed. The status of the TRPD was assessed by clinical (visual-tactile) and intraoral radiographic examination. The interval for the radiographic examination was defined on an individual basis. Assessment of the TRPD was done in the same practice by the dentist who prepared the TRPD when patients attended for routine care, recall, or when a problem occurred.

#### Success on TC-level

TC were provided without any technical complication (loss of retention of the dentures, decementation of the primary crowns, framework fractures, fractures of the dentures or prosthetic teeth, and chipping of the veneering resin composite) until the last follow-up examination. Additionally, no abutment tooth loss and no repairable defects occurred, such as abutment tooth fracture, which was, e.g., repairable with post and core build-up. No adaptations of the denture were necessary and the denture stability was not rated as insufficient [4].

Whenever a technical complication was observed or scheduled for one of these treatments at the last check-up — for which an appointment was then made with the patient — the intervention was considered as failed (primary end point: no success).

#### Success on TRPD-level

On TRPD-level, two different scenarios were included. Firstly, the failure of the first telescopic crown of a TRPD was considered as failure of the whole TRPD (“worst-case scenario”). Consequently, when all TC of a TRPD were defined as success, the TRPD was also defined as success. Expected interventions such as relining of the denture base or removal of sore spots were not classified as failures. Secondly, the failure of the last telescopic crown of a TRPD was considered as failure of the whole TRPD (“best-case scenario”). Consequently, when all TC of a TRPD were defined as failure, the TRPD was also defined as failure.

#### Survival on TC-level

When the TC were still in function at the last check-up visit and found to be clinically acceptable, the intervention was defined as survival even though an endodontic retreatment was needed in the meantime. Consequently, recementation (*n* = 39) or endodontic treatment (*n* = 36) was not considered as censored or failed. Contrastingly, the treatment was rated as failure (secondary end point: no survival) whenever a TC was renewed or the tooth was extracted [4].

#### Survival on TRPD-level

On TRPD-level, two different scenarios were included. Firstly, the failure of the first telescopic crown of a TRPD was considered as failure of the whole TRPD (“worst-case scenario”). Consequently, when all telescopic crowns of a TRPD survived, the TRPD survived. Secondly, the failure of the last telescopic crown of a TRPD was considered as failure of the whole TRPD (“best-case scenario”). Consequently, when all TC of a TRPD were defined as failure, the TRPD was also defined as failure.

### Statistical analysis and power analysis

For descriptive purposes, frequencies and percentages of measured baseline characteristics as well as frequencies and percentages of different failure types were tabulated.

Statistical analysis was performed using SPSS (SPSS 26.0; SPSS, Munich, Germany). Time until any failure was the dependent variable. Kaplan–Meier statistics and log-rank tests were used to calculate significant differences between the groups (*p* < 0.05). For Kaplan–Meier statistic, the independent method was used to generate success curves [16]. The annual failure rates (AFR) were calculated from life tables [22].

Crude associations between baseline characteristics and time until failure were calculated by fitting separate models for each baseline characteristic as the independent variable. Factors associated with time until failure (*p* < 0.25 [19, 23]) in the separate models were entered in a non-clustered multivariate Cox regression model (independent model).

For the present study, no prospective power or sample size calculation was performed. Regarding a retrospective power analysis for categories being included in multivariate Cox regression analysis, the analysis provided a power of ≥ 80% for none of the categories. Consequently, due to the pragmatic design of the present study, the study is likely to be underpowered to detect moderate to clinically significant relative risks in some categories.

## Results

Between April 2004 and March 2016, 174 telescopic dentures including 488 telescopic crowns in 139 patients were placed by two dentists. At the start of the observation period, patients’ mean age (SD) was 66 (11) years (min: 29; max 85). All primary and secondary crowns were conventionally cast from a non-precious alloy (Wironit LA, BEGO Bremer Goldschlägerei, Germany). The mean number of TC (standard deviation [SD]) per TRPD per patient was 2.8 (1.6) (range: 1–9). The mean observation time was 4.2 (3.3) years (maximum: 12 years) Characteristics of teeth are shown in Table 1 and Appendix Table 3.

**Table 1:** Frequency, number of failures of teeth included in study, and bivariate Cox proportional hazard regression analyses of time until failure (success) by categories of each baseline characteristic

Category		Patients*			Teeth										AFR_5years_ (%)
		Frequency (*n* (%))*	Failures (*n* (%))*		Frequency (*n* (%))		Failures (*n* (%))	*p*-value	HR	95% CI		Estimated median success time	95% CI		
Type of denture support															
Point-like		27 (16%)		7 (26%)		28 (6%)	7 (25%)		1.0	Reference		106.6	84.3–129		**6.5**
Linear		65 (39%)		21 (32%)		134 (27%)	33 (25%)	0.740	1.1	0.5–2.6		99.3	**89**.2–109.5		**5.5**
Triangle		31 (19%)		9 (%)		96 (20%)	16 (17%)	0.427	0.7	0.3–1.7		98.6	90.6–106.6		**3.4**
Rectangle		42 (25%)		18 (%)		230 (47%)	60 (26%)	0.540	1.3	0.6–2.8		91.5	84.4–98.7		**5.3**
Overall		165 (100%)		55 (33%)		488 (100%)	116 (24%)					103.3	97.6–109		**5.0**
Tooth type															
Incisor		41 (15%)		16 (39%)		98 (20%)	29 (30%)		1.0	Reference		87.3	77.1– 97.5		**6.1**
Canine		111 (41%)		31 (28%)		192 (39%)	45 (23%)	0.299	0.8	0.5–1.2		95.7	88.2–103.3		**4.8**
Premolar		91 (34%)		27 (%)		167 (34%)	36 (22%)	**0.110**	0.7	0.4–1.1		109.2	100–118.4		**5.1**
Molar		25 (9%)		5 (%)		31 (6%)	6 (19%)	0.305	0.6	0.3–1.5		95.8	80.1–111.5		**3.0**
Overall		268 (100%)		79 (29%)		488 (100%)	116 (24%)					103.3	97.6–109		**5.0**
Sex															
Female		66 (43%)		23 (35%)	286 (59%)		62 (22%)		1.0	Reference		107.8		100.6–114.9	**4.7**
Male		88 (57%)		29 (33%)	202 (41%)		54 (27%)	**0.051**	1.4	1–2.1		94.1		85.6–102.7	**5.6**
Overall		154 (100%)		52 (34%)	488 (100%)		116 (24%)					103.3		97.6–109	**5.0**
Insurance															
Statutorily		132 (85%)		41 (31%)	374 (77%)		78 (21%)		1.0	Reference		106.6		100–113.1	**4.6**
Privately		23 (15%)		12 (52%)	114 (23%)		38 (33%)	**0.108**	1.4	0.9–2		90.6		81.7–99.6	**6.3**
Overall		155 (100%)		53 (34%)	488 (100%)		116 (24%)					103.3		97.6–109	**5.0**
Number of abutment teeth															
1		23 (19%)	5 (22%)		22 (5%)		5 (23%)		1.0	Reference		108.1		82.7–133.6	**5.8**
2		54 (44%)	14 (26%)		108 (22%)		17 (16%)	0.662	0.8	0.3–2.2		109.7		98.4–120.9	**4.2**
3		29 (24%)	10 (34%)		87 (18%)		19 (22%)	0.903	0.9	0.4–2.5		93.9		85.1–102.8	**4.7**
4		16 (13%)	6 (38%)		61 (13%)		14 (23%)	0.465	1.5	0.5–4.1		81.9		69.6–94.2	**6.4**
≥ 5		33 (27%)	17 (52%)		210 (43%)		61 (29%)	0.551	1.3	0.5–3.3		91.4		84.5–98.4	**5.2**
Overall		122 (100%)	35 (29%)		488 (100%)		116 (24%)					103.3		97.6–109	**5.0**
Number of abutment teeth (dichotomized)															
≤ 3		120 (72%)	33 (28%)		262 (54%)		51 (19%)		1.0	Reference		111.0		103.6–118.4	**4.3**
> 3		46 (28%)	22 (48%)		226 (46%)		65 (29%)	**0.007**	1.7	1.1–2.4		88.6		81.3–95.8	**5.9**
Overall		166 (100%)	55 (33%)		488 (100%)		116 (24%)					103.3		97.6–109	**5.0**
Patients’ age															
> 61		88 (57%)	30 (34%)		268 (55%)		59 (22%)		1.0		Reference	100.0		92.3–107.6	**6.8**
31–60		61 (39%)	20 (33%)		202 (41%)		50 (25%)	0.373	0.8		0.6–1.2	105.1		97–113.3	**3.2**
< 30		6 (4%)	2 (33%)		18 (4%)		7 (39%)	0.757	1.1		0.5–2.5	97.8		75.1–120.5	**4.8**
Overall		155 (100%)	52 (34%)		488 (100%)		116 (24%)					103.3		97.6–109	**5.0**
Dentist															
1	127 (81%)		48 (38%)		412 (84%)		104 (25%)		1.0		Reference	103.4		97.4–109.4	**5.2**
2	29 (19%)		5 (17%)		76 (16%)		12 (16%)	0.969	1.0		0.6–1.9	73.4		65.9–80.8	**4.0**
Overall	156 (100%)		53 (34%)		488 (100%)		116 (24%)					103.3		97.6–109	**5.0**
DMFT															
≥ 21	98 (63%)		39 (40%)		327 (67%)		89 (27%)		1.0		Reference	No statistics are computed because all cases are censored in category “ ≤ 10”			**6.7**
20–11	51 (33%)		13 (25%)		151 (31%)		26 (17%)	**0.000**	0.4		0.3–0.7				**2.4**
≤ 10	1 (1%)		1 (100%)		2 (0%)		0 (0%)	0.960	0.0		n/a				**0.0**
n/a	5 (3%)		1 (20%)		8 (2%)		1 (13%)	**0.086**	0.2		0–1.3				**0.0**
Overall	155 (100%)		54 (35%)		488 (100%)		116 (24%)								**5.0**
Number of remaining own teeth (including teeth with telescopic crowns)															
1	4 (3%)		1 (25%)		4 (1%)		1 (25%)		1.0		Reference	69.0		13.6–124.4	**10.7**
2	12 (8%)		5 (42%)		24 (5%)		8 (33%)	0.854	1.2		0.2–9.7	79.1		60.7–97.6	**5.2**
3	14 (9%)		2 (14%)		39 (8%)		4 (10%)	0.383	0.4		0–3.4	101.1		90.2–112.1	**3.4**
4	15 (9%)		6 (40%)		54 (11%)		12 (22%)	0.820	0.8		0.1–6.1	93.1		78.7–107.4	**5.8**
≥ 5	159 (100%)		55 (35%)		365 (75%)		91 (25%)	0.820	0.8		0.1–5.7	103.0		96.6–109.5	**5.0**
Overall	159 (100%)		55 (35%)		486 (100%)		116 (24%)					0.0		0.0	**5.0**
Number of remaining own teeth (excluding teeth with telescopic crowns)															
0	48 (28%)		17 (35%)		166 (34%)		49 (30%)		1.0		Reference	79.2		71.7–86.7	**7.2**
1	11 (6%)		3 (27%)		45 (9%)		8 (18%)	**0.026**	0.4		0.2–0.9	95.9		88.9–102.8	**0.0**
2	12 (7%)		4 (33%)		34 (7%)		6 (18%)	**0.071**	0.5		0.2–1.1	103.4		88.5–118.2	**4.9**
3	12 (7%)		3 (25%)		36 (7%)		8 (22%)	0.293	0.7		0.3–1.4	90.5		74.5–106.5	**4.4**
4	23 (13%)		9 (39%)		54 (11%)		14 (26%)	**0.103**	0.6		0.3–1.1	98.1		85.7–110.6	**4.1**
≥ 5	66 (38%)		22 (33%)		153 (31%)		31 (20%)	**0.005**	0.5		0.3–0.8	111.5		102.1–121	**5.2**
Overall	172 (100%)		58 (34%)		488 (100%)		116 (24%)					103.3		97.6–109	**5.0**
Tooth position in the arch															
More mesial toot	92 (47%)		25 (27%)		208 (43%)		49 (24%)		1.0		Reference	109.8		102.2–117.4	**3.7**
Most distal tooth	102 (53%)		37 (36%)		160 (33%)		52 (33%)	**0.017**	1.6		1.1–2.4	92.7		83.6–101.8	**7.3**
Only tooth	31 (16%)		9 (29%)		41 (8%)		11 (27%)	**0.143**	1.6		0.8–3.1	83.6		68.9–98.2	**5.7**
n/a	35 (18%)		3 (9%)		79 (16%)		4 (5%)	0.539	0.7		0.3–2	100.8		84.9–116.7	**1.8**
Overall	194 (100%)		62 (32%)		488 (100%)		116 (24%)					103.3		97.6–109	**5.0**
Jaw															
Upper jaw	85 (49%)		28 (33%)		281 (122%)		63 (22%)		1.0		Reference	102.5		95.7–109.2	**4.1**
Lower jaw	89 (51%)		31 (35%)		207 (90%)		53 (26%)	0.310	1.2		0.8–1.7	100.4		91.4–109.3	**6.3**
Overall	174 (100%)		59 (34%)		488 (100%)		116 (24%)					103.3		97.6–109	**5.0**

^*^Starting and ending point were recorded on tooth-level. Thus, on patient-level, one patient may be listed in two subcategories

Factors associated with time until failure (*p* < 0.25; bold) in the separate models were entered in the multivariate Cox regression model (Table 2)

### Success and survival

**Fig. 1:**
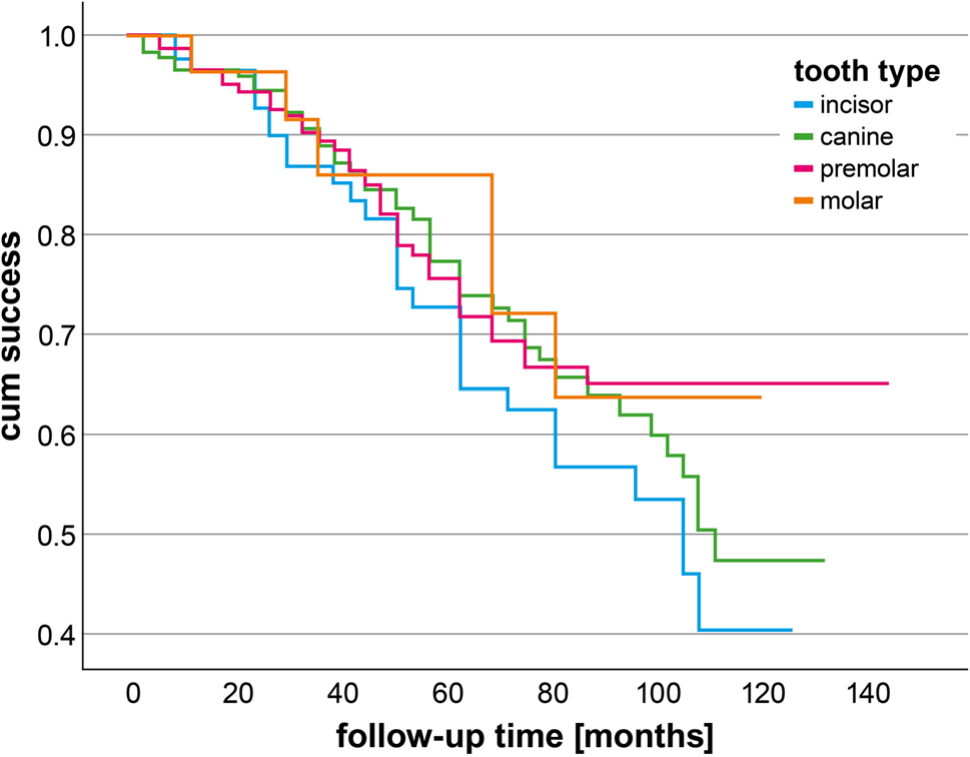
Kaplan-Meier curve for telescopic crowns according to the factor tooth type

**Fig. 2:**
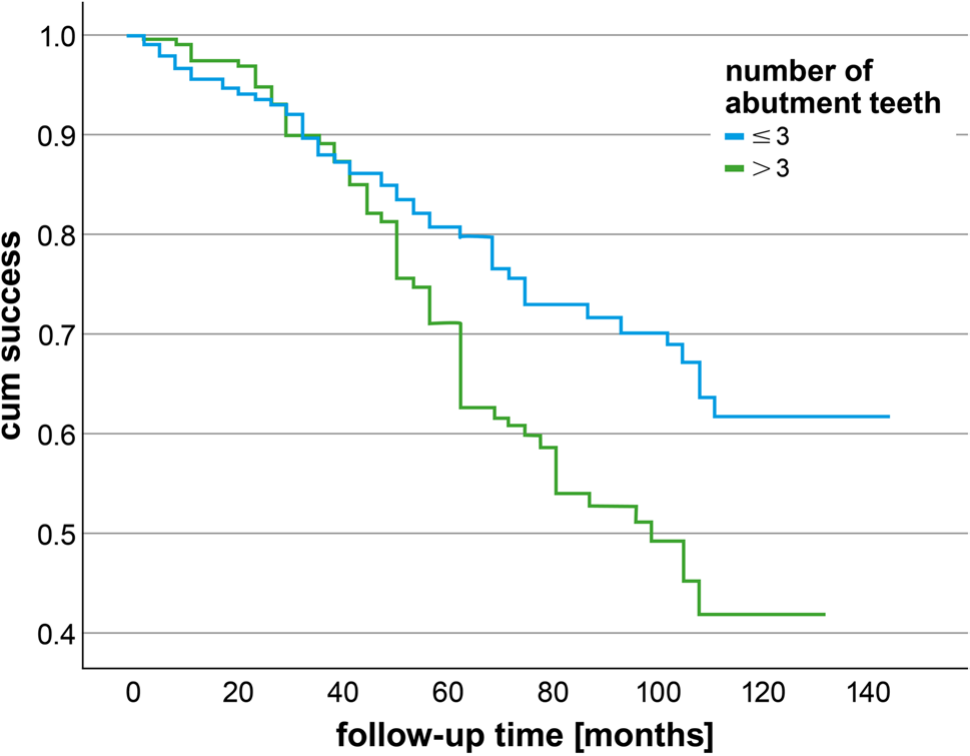
Kaplan-Meier curve for telescopic crowns according to the factor number of abutment teeth

Seventy-six percent of the telescopic crowns (372 out of 488) were considered as successful (Table 1) and 89% survived (433 out of 488) (Appendix Tables 3–4). The overall annual failure rate after 5 years of observation (AFR_5years_) for all dentists and restorations was 5.0% and 1.6%, respectively. The main failure types were recementation of the TC (*n* = 39), endodontic treatment (*n* = 36), and extraction of the tooth (*n* = 35). The success curves of restorations according to the tooth type are shown in Fig. 1. Success stratified according to number of abutment teeth are presented in Fig. 2.

On TRPD-level, 78% (136 out of 174) (“worst-case scenario”; Appendix Tables 5–6) and 86% (150 out of 174) (“best-case scenario”; Appendix Tables 7–8) of the dentures were considered as successful. Consequently, overall AFR_5years_ for all dentists and dentures were 5.1% and 3.4% respectively.

### Cox regression analysis 

Crude bivariate associations between the different baseline characteristics and an increased failure rate are given in Table 1 (success) and Appendix Table 3 (survival). Tooth type, patient’s sex, denture support type, DMFT, the number of remaining own teeth, and the position of the most distal TC (tooth position) were possibly associated with increased failure rates (*p* < 0.25).

The results of the non-clustered multivariate models including factors possibly associated with an increased failure rate in the bivariate models are shown in Table 2 (tooth-level scenario) and Appendix Table 4 (tooth-level scenario). The multivariate regression analysis showed that telescopic crowns in male patients showed 1.6 times (HR (95%CI): 1.596 (1.1–2.4); *p* = 0.023) higher risk for failure than telescopic crowns in female patients. Furthermore, telescopic crowns on premolars showed 2.2 times (HR: 1.908 (1.1–5.0); *p* = 0.015) lower risk for failure than on incisors. Telescopic crowns in dentures with ≤ 3 abutment teeth showed 2.1 times (HR: 2.075 (1.3–3.4); *p* = 0.004) lower risk for failure than telescopic crowns in dentures with > 3 abutment teeth. The position of the TC in the arch was a significant predictor as well. Telescopic crowns on the most distal tooth in an arch showed 2.4 times (HR: 2.429 (1.5–3.8); *p* < 0.001) higher risk for failure than telescopic crowns on a more mesial tooth.

**Table 2:** Multivariate Cox proportional hazard regression analyses of time until failure (success) as function of baseline characteristics identified

Category	*p*-value	HR	95% CI
Tooth type	0.092		
Incisor		1.0	Reference
Canine	0.189	0.684	0.4–1.2
Premolar	**0.015**	0.451	0.2–0.9
Molar	0.967	0.975	0.3–3.2
Patient’s sex			
Female		1.0	Reference
Male	**0.023**	1.596	1.1–2.4
Insurance			
Statutorily		1.0	Reference
Privately	0.964	1.011	0.6–1.6
Number of abutment teeth			
≤ 3		1.0	Reference
> 3	**0.004**	2.075	1.3–3.4
DMFT	**0.000**		
≥ 21		1.0	Reference
20–11	**0.000**	0.355	0.2–0.6
≤ 10	0.962	0.000	0–4.77 *10^191^
n/a	0.202	0.266	0–2
The number of remaining own teeth (excluding teeth with telescopic crowns)	**0.041**		
0		1.0	Reference
1	0.125	0.535	0.2–1.2
2	**0.012**	0.322	0.1–0.8
3	0.241	1.674	0.7–4
4	0.725	1.135	0.6–2.3
≥ 5	0.488	1.226	0.7–2.2
Tooth position in the arch	**0.001**		
More mesial tooth		1.0	Reference
Most distal tooth	**0.000**	2.429	1.5–3.8
Only tooth	0.300	1.512	0.7–3.3

Bold *p-*values (*p* < 0.05) indicate factors strongly associated with a de- or increased failure rate

Predictors being significant for success were also significant for survival (Appendix Table 4). Risk factors on TRPD levels for both scenarios can be found in Appendix Tables 6 and 8. However, from a statistical point of view, the number of TRPDs has to be considered as rather low. Therefore, these results should be interpreted with caution.

## Discussion

This single-center, practice-based, clinical cohort study retrospectively analyzed the survival and success of TRPDs and corresponding telescopic crowns. A total of 174 TRPDs retained by 488 non-precious alloy telescopic crowns by conventional casting technique were followed up for up to 12 years and the influence of several baseline parameters on the survival and success of the telescopic crowns was analyzed. At overall moderate annual failure rates, patient’s sex, DMFT, tooth type, tooth position, the number of abutment teeth, and the overall number of remaining own teeth were significant predictors for failure.

In the present study, the success rate of telescopic crowns was 76% with a mean follow-up time of 4.2 years. This result is very similar compared to other studies [4, 24]. Strasding et al. followed up in a similar study design at a university clinic, non-precious alloy telescopic crowns [4]. In the group with a mean follow-up time of 4.3 years, they showed a success rate of 77%. Similar to the present study, one of the main complications was the loss of abutment tooth sensitivity. The high frequency of recementations in the current study was also described in another university-based study [24].

In the present study, the longevity of the telescopic crowns was significantly affected by the patient’s sex. This is in agreement with previous studies of the AZT [19, 23] and with a previous university-based study on risk factors for tooth loss in clasp-retained RPD-wearers, [25]. Similar to the present study, the risk of failure was about two times higher in men compared to women. The authors attributed the elevated failure risk in men, to the higher probability of periodontal disease and the less frequent use of dental floss [25]. If one considers that men use dental floss less frequently than women, this indicates that oral hygiene may be less well established. This could further explain another finding of the present study, the correlation of a high DMFT value with the higher failure rates. Another factor that may lead to the higher failure rates in men is their higher chewing forces during mastication (as discussed below) [26].

The masticatory forces could also be the decisive factor why TRPDs in which the most posterior tooth of the jaw was an abutment tooth also showed higher rates of abutment tooth loss, in the present study [26]. Recently, it was observed that free-end partial denture wearers have lower maximum bite forces than denture wearers with abutments in terminal positions [27]. Since the highest masticatory forces occur in posterior jaw regions, it seems logical that teeth weakened by invasive preparation for a TC have an increased likelihood of loss due to the high loads. Tooth position as significant predictor for failure of telescopic crowns has also been described in another study [28]. After a mean observation time of 6.3 years, TRPDs in posterior abutment teeth were lost more frequently than in anterior teeth [28]. However, the authors did not mention any explanation for their observation. In addition to the aforementioned similarities with the existing literature, the lower incidence of tooth loss in premolars as abutment teeth of RPDs was also demonstrated before. Nisser et al. described in their study exactly the same HR of 2.2 for the loss of premolars compared to incisors as shown in the present study [25]. Considering these parallels, there seems to be no difference between telescopic crowns fabricated in a university or a private practice setting in terms of success and the type of complications.

However, not only parallels to the existing literature were found in the present study. For instance, the observed higher failure rate in TRPDs with more than 3 abutments contradicts the results from the existing literature. Several previous studies have observed lower failure rates when the TRPDs were retained on more than 3 abutment teeth [29–31]. The contradictory results might be explained with the used criteria about the preservation (success) or extraction (failure) of potential abutment teeth [32]. In the aforementioned (prospective) university-based studies, the criteria have been very strongly established, whereas in the present study, the criteria may have been less strictly established or followed, since a long-established dentist-patient relationship in a private practice is likely to result in a less aggressive treatment approach. Consequently, this may lead to the integration of questionable teeth into a removable prosthetic restoration in a private practice setting (which would have been extracted before a start of a prospective (university-based) study), especially if it is as easy to adapt for tooth losses as a TRPD. The combination of both, less aggressive treatment decisions and easily adaptable TRPD, could explain the higher failure rate in TRPDs with more than 3 abutment teeth in the present study.

Previous studies on direct and indirect restorations observed the importance of the factor dentist for restoration survival [18, 22, 33, 34]. As discussed previously, operator skills, “dentist profiles,” practice organization, and/or different patient needs may influence the longevity of dental treatments. This could also be seen in a study on root-cap retained RPDs analyzed the frequency of technical and biological complications manufactured in the pre-graduate and post-graduate education [35]. Despite a standardized clinical concept, a high degree of uniformity, and a supervised pre-graduate course, there were significantly more complications in the pre-graduate than in the post-graduate scenario. In order to (almost) exclude the structural factors of a private clinic (practice organization and/or different patient needs), all cases in the present study were treated in the same private clinic. Furthermore, treatment decisions and processes of the two dentists were harmonized due to the personal interaction (e.g., regular discussion of cases). This can presumably be seen in the results of the present study. Although no study dependent intra- or inter-examiner calibration regarding treatment decisions was performed prior to the study, the longevity of the TC was not significantly affected by the dentists and the AFR of the two dentists varied only slightly (5.8% vs. 5.5%). Nevertheless, the factor dentist should be subject of future studies on telescopic crowns.

Regarding success any invasive retreatment of the primary or secondary crown was classified as failure. Thus, even in the case of an endodontic treatment of the tooth, the respective TC was classified as failure since the primary crown received a filling on the occlusal/oral surface. This definition — as well as the definition of survival and failure — was based on previous practice-based studies [17, 18, 22, 33, 36–38]. Furthermore, the longevity of the primary crown is presumable weekend due to the endodontic treatment [39]. Nonetheless, a failure because of an endodontic treatment does not necessarily need to be directly connected to a failure of a TC. There are several other indications for an endodontic treatment. Unfortunately, the present dataset only presented the information that an endodontic treatment was done but not the indication for it. It remains, therefore, unclear if the endodontic treatment was or was not connected to the TC. Consequently, two options remained: classifying all endodontic treatments as “no failure” or as “failure.” In the case of “no failure,” the AFR would be underestimated (best-case scenario); in the case of “failure,” the AFR would be overestimated (worst-case scenario). Based on the previous studies [17, 18, 22, 33, 36–38], the worst-case scenario was chosen and even in this scenario, we observed moderate annual failure rates.

In the present study, two different perspectives to classify a failure on TRPD-level were used. On the one hand, in the “best-case scenario,” the failure of the last telescopic crown of a TRPD was considered as failure of the whole TRPD — as done previously [4]. Thus, it was taken into account that TRPD still “work” even when one (or more) telescopic crown is lost. On the other hand, in the “worst-case scenario,” the failure of the first telescopic crown of a TRPD was considered as failure of the whole TRPD. Thus, the cost-perspective was also taken into account, since each additional abutment tooth generates additional costs. These costs have to be payed either by an insurance or the patient himself. However, it is difficult to convince a patient that the original treatment was still successful even if he has lost one (or more) abutment teeth and thus money — especially if the patient bears the additional costs. Interestingly, in both scenarios, the risk factors were the same. In the present study, neither special protocols for the indication of a TRPD nor for the manufacturing process were performed. Furthermore, although the patient files were accurately and precisely documented, it was impossible to access all necessary information [19]. Furthermore, prophylactic treatments and oral health instructions were applied on an individual decision and not standardized (further information on shared decision-making were described [19]). No intra-examiner calibration with, e.g., respect to failure, success/survival as well as treatment decisions/processes, reintervention, and documentation resulting in adequate reproducibility prior to the study, was performed. This may cause difficulties to control bias and confounders. However, in the practice-based network, treatment decisions and processes are coordinated to evaluate and compare the quality of work as well as to receive feedback from colleagues [40]. The success of this kind of “examiner calibration” can presumably also be seen in the results of the present study. The longevity of the TC (and TRPD) was not significantly affected by the dentists. Thus, the present study setting not only reflects the real clinical situation but is also closer to daily clinical routine in dental practices than university-based studies.

Due to the retrospective design, no prospective power analysis was feasible in the present study. This is one major limitation, since the retrospective power analysis revealed that the present study may be underpowered to detect moderate to clinically significant relative risks. Consequently, it might be speculated that with a larger sample size or with more failures, the influence of some factors as (significant) predictor and the reliability of the present results would increase [17]. However, approximately 37134 TC would need to be included, for example, to provide a power of 80%, considering an *α*-error of 25% (bivariate analysis) and a median success times of, e.g., the upper (102.5 months) and lower (100.4 months) jaws. Furthermore, only two previous university-based studies on the longevity of TRPD included more dentures than the present study [3, 13], which makes the present study the largest one, reporting data from private practices. In contrast, most of the studies included less than the present 174 TRPD [3, 7–9, 11, 12, 14, 15].

Within the limitation of this study, moderate failure rates for removable partial dentures retained by telescopic crowns could be found in a private practice environment after up to 12 years. Patient- and tooth-level factors were significantly associated with failure. Telescopic crowns in male patients, telescopic crowns on premolars, and telescopic crowns in dentures with 4 or more telescopic crowns were significant predictors for failure. However, for telescopic crowns, further studies are needed to improve our knowledge about several of these factors and to analyze patient-related factors which have not been included in the present study.

## Data Availability

All data generated or analyzed during this study are included in this article (and/or) its supplementary material files. Further enquiries can be directed to the corresponding author.

## Appendix

**Table 3:** Frequency, number of failures of teeth included in study and bivariate Cox proportional hazard regression analyses of time until failure (survival) by categories of each baseline characteristic

	Teeth						
Category	Frequency [*n* (%)]	Failures [*n* (%)]	*p*-value	HR	95% CI	Estimated Median success time	95% CI
Type of denture support							
Point-like	28 (6%)	4 (14%)		1.0	Reference	123.3	106.4 − 140.2
Linear	134 (27%)	18 (13%)	0.624	1.3	0.4 − 3.9	114.5	105.7 − 123.3
Triangle	96 (20%)	5 (5%)	0.286	0.5	0.1 − 1.8	110.4	104.8 − 116
Rectangle	230 (47%)	28 (12%)	0.841	1.1	0.4 − 3.2	112.3	106.9 − 117.8
Overall	488 (100%)	55 (11%)				122.6	117.9 − 127.4
Tooth type							
Incisor	98 (20%)	14 (14%)		1.0	Reference	107.9	100.1 − 115.7
Canine	192 (39%)	23 (12%)	0.703	0.9	0.5 − 1.7	111.6	105.5 − 117.7
Premolar	167 (34%)	15 (9%)	**0.187**	0.6	0.3 − 1.3	128.1	121 − 135.1
Molar	31 (6%)	3 (10%)	0.670	0.8	0.2 − 2.7	107.8	96.1 − 119.4
Overall	488 (100%)	55 (11%)				122.6	117.9 − 127.4
Patient`s sex							
Female	286 (59%)	30 (10%)		1.0	Reference	124.8	119.1 − 130.5
Male	202 (41%)	25 (12%)	**0.169**	1.5	0.9 − 2.5	115.6	108.2 − 123
Overall	488 (100%)	55 (11%)				122.6	117.9 − 127.4
Insurance							
Statutorily	374 (77%)	38 (10%)		1.0	Reference	122.7	117 − 128.3
Privately	114 (23%)	17 (15%)	0.980	1.0	0.6 − 1.8	113.1	106.4 − 119.9
Overall	488 (100%)	55 (11%)				122.6	117.9 − 127.4
Number of abutment teeth							
1	22 (5%)	2 (9%)		1.0	Reference	130.0	114.3 − 145.7
2	108 (22%)	9 (8%)	0.706	1.3	0.3 − 6.2	121.1	111.7 − 130.6
3	87 (18%)	6 (7%)	0.985	1.0	0.2 − 4.9	108.9	102.7 − 115.1
4	61 (13%)	11 (18%)	**0.056**	4.4	1 − 20.2	87.1	75.7 − 98.5
≥ 5	210 (43%)	27 (13%)	0.569	1.5	0.4 − 6.4	113.8	108.8 − 118.9
Overall	488 (100%)	55 (11%)				122.6	117.9 − 127.4
Number of abutment teeth (dichotomized)							
≤ 3	262 (54%)	25 (10%)		1.0	Reference	125.4	119.3 − 131.6
> 3	226 (46%)	30 (13%)	**0.231**	1.4	0.8 − 2.4	111.0	105.3 − 116.6
Overall	488 (100%)	55 (11%)				122.6	117.9 − 127.4
Patients’ age							
> 61	268 (55%)	27 (10%)		1.0	Reference	119.1	113.1 − 125.2
31−60	202 (41%)	23 (11%)	0.771	0.9	0.5 − 1.6	123.7	116.8 − 130.7
< 30	18 (4%)	5 (28%)	0.465	1.4	0.5 − 3.7	117.0	103.4 − 130.6
Overall	488 (100%)	55 (11%)				122.6	117.9 − 127.4
Dentist							
1	412 (84%)	52 (13%)		1.0	Reference	122.3	117.4 − 127.2
2	76 (16%)	3 (4%)	0.607	0.7	0.2 − 2.4	83.6	77.4 − 89.8
Overall	488 (100%)	55 (11%)				122.6	117.9 − 127.4
DMFT							
≥ 21	327 (67%)	42 (13%)		1.0	Reference	No statistics are computed because all cases are censored in category “≤10”	
20−11	151 (31%)	12 (8%)	**0.015**	0.4	0.2 − 0.9		
≤ 10	2 (0%)	0 (0%)	0.969	0.0	0 − 1.0148837412096E+248		
n/a	8 (2%)	1 (13%)	0.290	0.3	0 − 2.5		
Overall	488 (100%)	55 (11%)					
Number of remaining own teeth (including teeth with telescopic crowns)							
1	4 (1%)	1 (25%)		1.0	Reference	88.0	58.9 − 117.1
2	24 (5%)	4 (17%)	0.928	0.9	0.1 − 8.1	96.4	79.8 − 113
3	39 (8%)	1 (3%)	**0.168**	0.1	0 − 2.3	107.3	97.2 − 117.5
4	54 (11%)	6 (11%)	0.493	0.5	0.1 − 4	110.7	101 − 120.5
≥ 5	365 (75%)	43 (12%)	0.476	0.5	0.1 − 3.5	122.8	117.4 − 128.1
Overall	486 (100%)	55 (11%)				0.0	0.0
Number of remaining own teeth (excluding teeth with telescopic crowns)							
0	166 (34%)	21 (13%)		1.0	Reference	102.3	97 − 107.7
1	45 (9%)	2 (4%)	**0.143**	0.3	0.1 − 1.4	103.0	98.6 − 107.4
2	34 (7%)	5 (15%)	0.923	1.0	0.4 − 2.5	107.6	94 − 121.2
3	36 (7%)	7 (19%)	**0.165**	1.8	0.8 − 4.3	92.5	76.8 − 108.3
4	54 (11%)	7 (13%)	0.771	0.9	0.4 − 2.1	112.2	101.8 − 122.6
≥ 5	153 (31%)	13 (8%)	**0.139**	0.6	0.3 − 1.2	128.4	121.1 − 135.8
Overall	488 (100%)	55 (11%)				122.6	117.9 − 127.4
Tooth position in the arch							
More mesial tooth	208 (43%)	17 (8%)		1.0	Reference	130.9	125.6 − 136.2
Most distal tooth	160 (33%)	27 (17%)	**0.003**	2.5	1.4 − 4.6	111.7	103.9 − 119.5
Only tooth	41 (8%)	8 (20%)	**0.001**	4.2	1.8 − 9.8	91.8	78.1 − 105.5
n/a	79 (16%)	3 (4%)	0.268	2.0	0.6 − 6.9	104.9	90.7 − 119.2
Overall	488 (100%)	55 (11%)				122.6	117.9 − 127.4
Jaw							
Upper jaw	281 (58%)	29 (10%)		1	Reference	121.2	116 − 126.4
Lower jaw	207 (42%)	26 (13%)	**0.198**	1.4	0.8 − 2.4	117.9	109.8 − 126

*Starting and ending point were recorded on tooth-level. Thus, on patient level one patient may be listed in two subcategories

Factors associated with time until failure (*p* < 0.25; bold) in the separate models were entered in the multivariate Cox regression model (Appendix Table 4)

**Table 4:** Multivariate Cox proportional hazard regression analyses of time until failure (survival) as function of baseline characteristics identified

Category	*p*-value	HR	95% CI
Tooth type	0.118		
Insicor		1.0	Reference
Canine	0.143	0.533	0.2 − 1.2
Premolar	**0.015**	0.287	0.1 − 0.8
Molar	0.665	0.661	0.1 − 4.3
Patient's sex			
Female		1.0	Reference
Male	**0.037**	1.925	1 − 3.6
Number of abutment teeth			
≤ 3		1.0	Reference
> 3	**0.011**	2.578	1.2 − 5.3
DMFT	0.380		
≥ 21		1.0	Reference
20-11	**0.004**	0.301	0.1 − 0.7
≤ 10	0.973	0.000	0 − 3.1338570248433E+263
n/a	0.728	0.684	0.1 − 5.8
The number of remaining own teeth (excluding teeth with telescopic crowns)	**0.004**		
0		1.0	Reference
1	0.584	0.656	0.1 − 3
2	0.946	1.038	0.4 − 3
3	**0.000**	9.389	3 − 29.2
4	**0.038**	3.295	1.1 − 10.1
≥ 5	0.091	2.400	0.9 − 6.6
Tooth position in the arch	**< 0.001**		
More mesial tooth		1.0	Reference
Most distal tooth	**< 0.001**	5.281	2.5 − 11.3
Only tooth	**< 0.001**	8.630	2.7 − 27.8
n/a	0.896	1.136	0.2 − 7.6
Jaw			
Upper jaw		1.0	Reference
Lower jaw	0.938	0.976	0.5 − 1.8

Bold *p*-values (*p* < 0.05) indicate factors strongly associated with a de- or increased failure rate

**Table 5:** Frequency, number of failures of removable partial dentures retained by telescopic crowns (TRPC) included in study and bivariate Cox proportional hazard regression analyses of time until failure (success) by categories of each baseline characteristic [“worst-case scenario”]

	Teeth						
Category	Frequency [*n* (%)]	Failures [*n* (%)]	*p*-value	HR	95% CI	Estimated Median success time	95% CI
Type of denture support							
Point-like	28 (16%)	7 (25%)		1.0	Reference	106.6	84.3 − 129
Linear	66 (38%)	15 (23%)	0.745	1.2	0.5 − 2.9	97.9	82.7 − 113.1
Triangle	32 (18%)	4 (13%)	0.374	0.6	0.2 − 2	102.1	89 − 115.2
Rectangle	48 (28%)	12 (25%)	0.466	1.4	0.6 − 3.6	83.8	69 − 98.7
Overall	174 (100%)	38 (22%)				104.2	94.3 − 114
Patient`s sex							
Female	101 (58%)	20 (20%)		1.0	Reference	108.2	96 − 120.5
Male	73 (42%)	18 (25%)	0.250	1.5	0.8 − 2.8	94.9	79.7 − 110.1
Overall	174 (100%)	38 (22%)				104.2	94.3 − 114
Insurance							
Statutorily	145 (83%)	29 (20%)		1.0	Reference	107.2	96.4 − 117.9
Privately	29 (17%)	9 (31%)	0.300	1.5	0.7 − 3.1	81.7	65.4 − 98
Overall	174 (100%)	38 (22%)				104.2	94.3 − 114
Number of abutment teeth							
1	22 (13%)	5 (23%)		1.0	Reference	108.1	82.7 − 133.6
2	55 (32%)	8 (15%)	0.625	0.8	0.2 − 2.3	108.8	92.1 − 125.5
3	32 (18%)	7 (22%)	0.959	1.0	0.3 − 3.3	92.4	77 − 107.8
4	17 (10%)	4 (24%)	0.486	1.6	0.4 − 6	80.3	55.3 − 105.2
≥ 5	48 (28%)	14 (29%)	0.475	1.5	0.5 − 4	85.0	72.1 − 97.9
Overall	174 (100%)	38 (22%)				104.2	94.3 − 114
Number of abutment teeth (dichotomized)							
≤ 3	127 (73%)	24 (19%)		1.0	Reference	110.5	99.7 − 121.4
> 3	47 (27%)	14 (30%)	**0.032**	2.1	1.1 − 4.1	72.6	59.9 − 85.3
Overall	174 (100%)	38 (22%)				104.2	94.3 − 114
Patients’ age							
> 61	99 (57%)	21 (21%)		1.0	Reference	102.1	89.6 − 114.7
31−60	68 (39%)	14 (21%)	0.635	0.8	0.4 − 1.7	106.0	91.2 − 120.9
< 30	7 (4%)	3 (43%)	0.460	1.6	0.5 − 5.3	86.5	48.5 − 124.6
Overall	174 (100%)	38 (22%)				104.2	94.3 − 114
Dentist							
1	145 (83%)	35 (24%)		1.0	Reference	103.4	93 − 113.8
2	29 (17%)	3 (10%)	0.662	0.8	0.2 − 2.5	70.8	62.8 − 78.8
Overall	174 (100%)	38 (22%)				104.2	94.3 − 114
DMFT							
≥ 21	113 (65%)	28 (25%)		1.0	Reference	No statistics are computed because all cases are censored in category “≤ 10”	
20−11	54 (31%)	9 (17%)	**0.034**	0.4	0.2 − 0.9		
≤ 10	1 (1%)	0 (0%)	0.972	0.0	0 − 7.8041344391003E+274		
n/a	6 (3%)	1 (17%)	**0.155**	0.2	0 − 1.7		
Overall	174 (100%)	38 (22%)					
Number of remaining own teeth (including teeth with telescopic crowns)							
1	4 (2%)	1 (25%)		1.0	Reference	69.0	13.6 − 124.4
2	12 (7%)	5 (42%)	0.634	1.7	0.2 − 14.6	68.2	42.4 − 94
3	14 (8%)	2 (14%)	0.696	0.6	0.1 − 6.9	95.8	74.3 − 117.3
4	15 (9%)	5 (33%)	0.926	1.1	0.1 − 9.6	87.3	61.8 − 112.8
≥ 5	127 (73%)	25 (20%)	0.732	0.7	0.1 − 5.2	107.8	96.5 − 119
Overall	172 (99%)	38 (22%)				104.2	94.3 − 114
Number of remaining own teeth (excluding teeth with telescopic crowns)							
0	46 (26%)	15 (33%)		1.0	Reference	73.2	58 − 88.4
1	11 (6%)	2 (18%)	**0.205**	0.4	0.1 − 1.7	94.8	74.5 − 115.1
2	12 (7%)	1 (8%)	**0.195**	0.3	0 − 2	108.8	81.2 − 136.3
3	11 (6%)	2 (18%)	0.673	0.7	0.2 − 3.2	62.9	44.4 − 81.4
4	23 (13%)	5 (22%)	**0.202**	0.5	0.2 − 1.4	97.6	78.5 − 116.7
≥ 5	71 (41%)	13 (18%)	**0.020**	0.4	0.2 − 0.9	113.3	99.6 − 127.1
Overall	174 (100%)	38 (22%)				104.2	94.3 − 114
Jaw							
Upper jaw	85 (177%)	18 (21%)		1.0	Reference	103.0	90.2 − 115.8
Lower jaw	89 (185%)	20 (22%)	0.691	1.1	0.6 − 2.2	102.7	88.7 − 116.6

*Starting and ending point were recorded on TRPC-level. Thus, on patient level one patient may be listed in two subcategories

Factors associated with time until failure (*p* < 0.25; bold) in the separate models were entered in the multivariate Cox regression model (Appendix Table 6)

**Table 6:** Multivariate Cox proportional hazard regression analyses of time until failure (success) as function of baseline characteristics identified [“worst-case scenario”]

Category	*p*-value	HR	95% CI
Number of abutment teeth			
≤ 3		1.0	Reference
> 3	**0.011**	2.578	1.2 − 5.3
Patients’ age	0.198		
> 61		1.0	Reference
41−60	0.648	0.849	0.4 − 1.7
< 40	0.107	3.484	0.8 − 15.9
DMFT	0.175		
≥ 21		1.0	Reference
20−11	0.076	0.477	0.2 − 1.1
≤ 10	0.974	0.000	0 − 3.8682284379951E+295
n/a	0.114	0.158	0 − 1.6
The number of remaining own teeth (excluding teeth with telescopic crowns)	0.350		
0		1.0	Reference
1	**0.059**	0.190	0 − 1.1
2	0.173	0.242	0 − 1.9
3	0.826	1.187	0.3 − 5.5
4	0.775	0.854	0.3 − 2.5
≥ 5	0.399	0.704	0.3 − 1.6

Bold *p*-values (*p* < 0.05) indicate factors strongly associated with a de- or increased failure rate

**Table 7:** Frequency, number of failures of removable partial dentures retained by telescopic crowns (TRPC) included in study and bivariate Cox proportional hazard regression analyses of time until failure (success) by categories of each baseline characteristic [“best-case scenario”]

	Teeth						
Category	Frequency [*n* (%)]	Failures [*n* (%)]	*p*-value	HR	95% CI	Estimated Median success time	95% CI
Type of denture support							
Point-like	28 (16%)	7 (25%)		1.0	Reference	106.6	84.3 − 129
Linear	66 (38%)	9 (14%)	0.453	0.7	0.3 − 1.8	111.7	97.3 − 126.1
Triangle	32 (18%)	2 (6%)	**0.118**	0.3	0.1 − 1.4	109.6	99.7 − 119.5
Rectangle	48 (28%)	6 (13%)	0.496	0.7	0.2 − 2.1	99.2	85.4 − 113
Overall	174 (100%)	24 (14%)				117.1	107.8 − 126.3
Patient`s sex							
Female	101 (58%)	11 (11%)		1.0	Reference	122.9	112 − 133.8
Male	73 (42%)	13 (18%)	**0.117**	1.9	0.9 − 4.3	104.1	89.1 − 119.1
Overall	174 (100%)	24 (14%)				117.1	107.8 − 126.3
Insurance							
Statutorily	145 (83%)	20 (14%)		1.0	Reference	117.4	107.3 − 127.4
Privately	29 (17%)	4 (14%)	0.942	1.0	0.3 − 2.8	97.5	81.9 − 113.1
Overall	174 (100%)	24 (14%)				117.1	107.8 − 126.3
Number of abutment teeth							
1	22 (13%)	5 (23%)		1.0	Reference	108.1	82.7 − 133.6
2	55 (32%)	3 (5%)	**0.082**	0.3	0.1 − 1.2	125.8	113.6 − 138
3	32 (18%)	5 (16%)	0.637	0.7	0.2 − 2.6	98.7	84.4 − 113.1
4	17 (10%)	2 (12%)	0.767	0.8	0.1 − 4.1	96.6	78.1 − 115.1
≥ 5	48 (28%)	9 (19%)	0.903	0.9	0.3 − 2.8	93.9	80.8 − 107
Overall	174 (100%)	24 (14%)				117.1	107.8 − 126.3
Number of abutment teeth (dichotomized)							
≤ 3	127 (73%)	17 (13%)		1.0	Reference	119.5	109.5 − 129.5
> 3	47 (27%)	7 (15%)	0.454	1.4	0.6 − 3.4	85.1	72.9 − 97.3
Overall	174 (100%)	24 (14%)				117.1	107.8 − 126.3
Patients’ age							
> 61	99 (57%)	16 (16%)		1.0	Reference	109.6	97.7 − 121.5
31−60	68 (39%)	7 (10%)	**0.211**	0.6	0.2 − 1.4	120.9	106.6 − 135.1
< 30	7 (4%)	1 (14%)	0.752	0.7	0.1 − 5.5	114.0	73.7 − 154.3
Overall	174 (100%)	24 (14%)				117.1	107.8 − 126.3
Dentist							
1	145 (83%)	22 (15%)		1.0	Reference	116.3	106.5 − 126
2	29 (17%)	2 (7%)	0.722	0.8	0.2 − 3.3	72.2	64.4 − 80
Overall	174 (100%)	24 (14%)				117.1	107.8 − 126.3
DMFT							
≥ 21	113 (65%)	17 (15%)		1.0	Reference	No statistics are computed because all cases are censored in category “20-11”	
20−11	54 (31%)	7 (13%)	**0.238**	0.6	0.2 − 1.4		
≤ 10	1 (1%)	0 (0%)	0.991	0.0	0 − 0		
n/a	6 (3%)	0 (0%)	0.980	0.0	0 − 0		
Overall	174 (100%)	24 (14%)					
Number of remaining own teeth (including teeth with telescopic crowns)							
1	4 (2%)	1 (25%)		1.0	Reference	69.0	13.6 − 124.4
2	12 (7%)	3 (25%)	0.967	1.0	0.1 − 9.2	81.9	56.4 − 107.3
3	14 (8%)	1 (7%)	0.391	0.3	0 − 4.8	104.9	89.7 − 120.2
4	15 (9%)	2 (13%)	0.497	0.4	0 − 4.9	108.5	87.8 − 129.2
≥ 5	127 (73%)	17 (13%)	0.461	0.5	0.1 − 3.5	118.3	107.8 − 128.8
Overall	172 (99%)	24 (14%)				0.0	0.0
Number of remaining own teeth (excluding teeth with telescopic crowns)							
0	46 (26%)	9 (20%)		1.0	Reference	No statistics are computed because all cases are censored in category “2”	
1	11 (6%)	1 (9%)	0.295	0.3	0 − 2.6		
2	12 (7%)	0 (0%)	0.982	0.0	0 − 0		
3	11 (6%)	2 (18%)	0.826	1.2	0.3 − 5.5		
4	23 (13%)	2 (9%)	**0.181**	0.4	0.1 − 1.6		
≥ 5	71 (41%)	10 (14%)	**0.185**	0.5	0.2 − 1.3		
Overall	174 (100%)	24 (14%)					
Jaw							
Upper jaw	85 (177%)	12 (14%)		1.0	Reference	112.9	100.9 − 124.9
Lower jaw	89 (185%)	12 (13%)	0.951	1.0	0.5 − 2.3	117.7	104.8 − 130.5

*Starting and ending point were recorded on TRPC-level. Thus, on patient level one patient may be listed in two subcategories

Factors associated with time until failure (*p* < 0.25; bold) in the separate models were entered in the multivariate Cox regression model (Appendix Table 6)

**Table 8:** Multivariate Cox proportional hazard regression analyses of time until failure (success) as function of baseline characteristics identified [“best-case scenario”]

Category	*p*-value	HR	95% CI
Type of denture support			
Point-like		1.0	Reference
Linear	0.563	0.727	0.2 − 2.1
Triangle	**0.101**	0.249	0 − 1.3
Rectangle	0.567	0.694	0.2 − 2.4
Patient's sex			
Female		1.0	Reference
Male	0.278	1.603	0.7 − 3.8
Patients’ age	0.547		
> 61		1.0	Reference
41−60	0.505	0.722	0.3 − 1.9
< 40	0.457	2.346	0.2 − 22.3
DMFT	0.685		
≥ 21		1.0	Reference
20−11	0.223	0.532	0.2 − 1.5
≤ 10	0.992	0.000	0 − 0
n/a	0.982	0.000	0 − 0
The number of remaining own teeth (excluding teeth with telescopic crowns)	0.754		
0		1.0	Reference
1	**0.330**	0.323	0 − 3.1
2	0.983	0.000	0 − 0
3	0.482	1.832	0.3 − 9.9
4	0.438	0.525	0.1 − 2.7
≥ 5	0.564	0.742	0.3 − 2

Bold *p*-values (*p* < 0.05) indicate factors strongly associated with a de- or increased failure rate
